# Discovery of A-type procyanidin dimers in yellow raspberries by untargeted metabolomics and correlation based data analysis

**DOI:** 10.1007/s11306-016-1090-x

**Published:** 2016-08-08

**Authors:** Elisabete Carvalho, Pietro Franceschi, Antje Feller, Lorena Herrera, Luisa Palmieri, Panagiotis Arapitsas, Samantha Riccadonna, Stefan Martens

**Affiliations:** Research and Innovation Centre, Fondazione Edmund Mach (FEM), Via E. Mach 1, 38010 San Michele all’Adige, Italy

**Keywords:** Metabolomics, Polyphenols, Secondary metabolism, *Rubus idaeus*, Data analysis

## Abstract

**Introduction:**

Raspberries are becoming increasingly popular due to their reported health beneficial properties. Despite the presence of only trace amounts of anthocyanins, yellow varieties seems to show similar or better effects in comparison to conventional raspberries.

**Objectives:**

The aim of this work is to characterize the metabolic differences between red and yellow berries, focussing on the compounds showing a higher concentration in yellow varieties.

**Methods:**

The metabolomic profile of 13 red and 12 yellow raspberries (of different varieties, locations and collection dates) was determined by UPLC–TOF-MS. A novel approach based on Pearson correlation on the extracted ion chromatograms was implemented to extract the pseudospectra of the most relevant biomarkers from high energy LC–MS runs. The raw data will be made publicly available on MetaboLights (MTBLS333).

**Results:**

Among the metabolites showing higher concentration in yellow raspberries it was possible to identify a series of compounds showing a pseudospectrum similar to that of A-type procyanidin polymers. The annotation of this group of compounds was confirmed by specific MS/MS experiments and performing standard injections.

**Conclusions:**

In berries lacking anthocyanins the polyphenol metabolism might be shifted to the formation of a novel class of A-type procyanidin polymers.

**Electronic supplementary material:**

The online version of this article (doi:10.1007/s11306-016-1090-x) contains supplementary material, which is available to authorized users.

## Introduction

The positive effects of a fruit and vegetable rich diet on human health are widely recognized. In the specific case of soft fruits, an increasing number of studies report on the beneficial effects of raspberries in terms of antioxidant activity (Wolfe et al. 2008), cancer prevention (Coates et al. 2007; Seeram 2008) and arthritis (Jean-Gilles et al. 2012). It is believed that these effects are related to the presence of high amounts of phenolic compounds. In the case of red varieties anthocyanins are undoubtedly the major player: red raspberries contain more than 110 mg/100 g FW of anthocyanins, the majority of which consists of cyanidin 3-*O*-sophoroside, with lower amounts of other cyanidin glycosides and even much lower amounts of pelargonidin glycosides (Mazur et al. 2014). Ellagitannins (sanguiin H-6 and lambertianin C) represent the second important class of phenolics, showing an average concentration of 100 mg/100 g FW (Gasperotti et al. 2010).

Raspberries come in a variety of colours, ranging from very pale yellow to almost purple. Yellow varieties, apart from the much lower amounts in anthocyanidins, seem to have similar compositions to red varieties in terms of total phenolic compounds (Anttonen and Karjalainen 2005; Carvalho et al. 2013; Weber et al. 2008). In one particular study, the phenol content of the yellow raspberry cultivar “Fallgold” was even reported to be higher than that of red cultivars such as “Tulameen” or “Autumn Bliss” (Pantelidis et al. 2007). Interestingly, even if they are almost completely lacking the anthocyanins, yellow raspberries seem to have similar or better health beneficial properties than red varieties. Both yellow and red raspberries were indeed equally able to inhibit alpha-amylase (Grussu et al. 2011), and yellow raspberry varieties showed antioxidant and antiproliferative activities (Liu et al. 2002). Moreover, yellow raspberry varieties were the most effective in inhibiting alpha-glucosidase and the most potential for ACE-1 inhibition, which could have important impact in diabetes and hypertension management (Cheplick et al. 2007). The presence of a similar phenol content in red and yellow varieties as well as the proposed health effects suggest that in yellow raspberries the metabolism might be shifted from the formation of anthocyanins to the formation of other type of phenolic compounds. Nevertheless, even though the phenolic composition of *Rubus* is very well characterized, and the color is one of the most important traits in these fruits, the genetic control of anthocyanin accumulation in raspberry is not yet well understood (Bushakra et al. 2013).

In the last few years, we have been interested in finding the position of the biochemical block responsible for the lack of anthocyanins in yellow raspberries and its influence on the overall phenolic profile of yellow varieties. In a previous targeted metabolic profile, we have shown that yellow raspberries contain lower amounts of a minor procyanidin dimer (B1), while other major procyanidins and phenolics remained unchanged (Carvalho et al. 2013). A question that arose from this result was if other compounds, particularly procyanidin polymers (trimers or higher), or compounds not detected in the targeted analytical method could be also altered in yellow raspberries. This type of information would be crucial to investigate the presence of a possible block in the pathway and, additionally, it would also give valuable information on the composition of yellow raspberries and on the compounds responsible of their interesting biological activity. As such, in the present study we used untargeted metabolomics to assess the differences between red and yellow raspberries. We analysed several red and yellow raspberry fruits, from different cultivars, locations and years, in order to find different compounds that are related to the anthocyanin biosynthesis block and to assess which other metabolic changes could be present in the yellow cultivars.

It is well known that in metabolomics the annotation of the results (i.e. the process of associating the different mass spectrometric features to the metabolites) is a major challenge and this process is even more difficult if it is not possible to take advantage of pure standards. In this case, the first step in the interpretation of the results is to extract the mass spectrum of the unknown compound from the untargeted dataset. In most of the cases this step is done by the analyst going back to the raw experimental data to check the feature elution profiles in the vicinity of the more “interesting” features. To partially automatize this process, we developed a new data analysis pipeline which uses a correlation based approach to identify the pseudospectra of the compounds showing a significant difference between yellow and red raspberries. In this study we first start by describing the complexity of the results and the data analysis pipeline approach. Then we discuss the differences found between the red and yellow raspberries (biomarkers) and based on the much richer information obtained in the form of pseudospectra, attempt to assign an identification to these markers. Finally, we confirm the validity of the “digitally” obtained pseudospectra by performing standard injections or performing MS/MS experiments. Based on exact mass and fragmentation patterns we propose that yellow raspberries have higher amounts of compounds that have similar characteristics to A-type procyanidins.

## Materials and methods

### Plant material

Raspberry fruits of different colors and varieties were collected from different locations as described in Table S1. The fruits were collected in the ripe stage (when the berry is easily detached from the crown). DR samples were collected in Julius Kühn Institute (Dresden, Germany), VG samples were collected from the the Berries Germplasm Collection of FEM, in Pergine Valsugana (Trento, Italy), BP samples were collected from the company Berry Plant (Verona, Italy). Samples were collected in different years as described in the Table S1. All samples collected in 2010 were stored in cooled containers and transported to FEM laboratories where they were flash frozen and stored. Samples collected in Dresden were frozen in dry ice and transported to FEM. All other samples were flash frozen with liquid nitrogen immediately after collection and transported in dry ice to FEM.

### Chemicals and solvents

Internal standards 3,5-hydroxybenzoic acid and 4-hydroxy stilbene as well as solvents acetone (ACS grade), methanol (LC–MS grade), and formic acid (LC–MS grade) were purchased from Sigma Aldrich (St. Louis, MO, USA). Water for chromatography and sample extraction was purified in a milli-Q device. The standard of procyanidin A2 was obtained from Extrasyntheses (Genay, FR).

### Analytical methods

#### Extraction

Small portions of each of the raspberry samples were mixed to prepare a quality control sample (QC). For each sample and QC, the extraction was made using a method adapted from Gasperotti et al. (2010): 12.0 ± 0.5 g of frozen raspberries were weighed in plastic 50 mL falcon tubes, 150 μg internal standard (3,5-hydroxybenzoic acid) and 10 mL of acetone 70 % were added to each tube. Samples were homogenized with an IKA Ultraturrax disperser (Staufen, Germany) for 1.5 min. 10 mL of acetone 70 % were used to help wash and collect all the sample from the homogenizer. The sample was allowed to extract for 20 min, after which was centrifuged for 5 min at 5000 rcf. The supernatant was collected into a 100 mL round bottom flask, filtering through glass wool to help collect any solid residues. Further 20 mL of acetone 70 % were added to the pellet, vortexed vigorously to help homogenization and centrifuged as before. The second (clear) supernatant was joined to the first one. After washing the funnel and glass wool with 5 mL of acetone 70 %, the acetone was removed by rotary evaporation at 40 °C. The concentrated extract was transferred to a 20 mL volumetric flask and brought to volume with 5 % methanol. 480 μL of each extract was transferred to an eppendorf containing 20 μL of 4-hydroxystilbene 5.6 g/L, filtered through 0.2 μm PTFE filters into 2 mL amber (LC–MS certificated) vials, and stored at 4 °C until the time of analysis. Samples were prepared in random order.

#### LC–MS

Liquid chromatography was performed on a Waters acquity UPLC equipped with an ACQUITY UPLC 1.8 μm 2.1 × 150 mm HSS T3 column. Detection was made on a SYNAPT HDMS QTOF MS, equipped with electrospray ionization (ESI) source, all from Waters (Manchester, UK). The instrument was controlled by the MassLynx 4.1 software provided. The LC and MS conditions used have been previously described (Theodoridis et al. 2012), with small differences. Data was collected in negative ion mode from 50 to 3000 *m/z*, from two different acquisition channels (high and low energy). The transfer collision energy and trap collision energy were set at 6 and 4 V for the low energy channel and 30 and 6 V for the high energy. All raspberry samples were randomized again and analyzed in negative mode. During the analysis, every six samples one QC was injected and every 12 samples one standard mix (STDMIX), prepared as described in (Theodoridis et al. 2012), was also injected. In the beginning one blank, one STDMIX and four QCs were injected. STDMIX, QCs and internal standards were used to assess the quality of the data as described in (Want et al. 2013). Raw LC–MS data will be made publicly available on MetaboLights (MTBLS333).

MS/MS experiments on the putative A-type procyanidins were performed by injecting selected yellow and red raspberries extracts on the same instrument using the modified chromatographic method described in (Arapitsas et al. 2014). Data was collected in negative ion V mode, from 80 to 2000 *m/z* using the same settings and collision energies as the LC–MS method to determine the new retention times. The parent ions 751.1, 1327.3, and 1039.2, 927.2 were selected in the quadrupole with non-overlapping time windows. The molecular ions were fragmented by setting the transfer collision energy to 20 V. The trap collision energies used were 20, 20, 15, and 30 V for each of the ions, respectively. The results of the MS/MS experiments are reported with lower accuracy due to the lower resolution obtained in the V mode.

### Data analysis

Raw HPLC–MS files were converted into open-source CDF format by using the DataBridge utility included in MassLynx4.1 from Waters. Feature extraction was performed by using the centWave algorithm (Tautenhahn et al. 2008) implemented within the R bioconductor package xcms (Smith et al. 2006). Full details about the data processing pipeline can be found in Table S2. Features having *m/z* greater than 2900 or retention time greater than 3000 s (50 min) or smaller than 30 s were excluded from the subsequent analysis. The feature list was annotated for adducts and common isotopes by the R bioconductor CAMERA package (Kuhl et al. 2012). At the end of this pre-processing phase a data matrix of 6365 *features* was extracted from the CAMERA S4 object.

The identification of the pseudospectra associated to the biomarkers from the full dataset is one of the major challenges in untargeted metabolomics. The statistical analysis is indeed performed on a “feature based” data matrix, while to putatively identify the “biomarkers” it is necessary to reconstruct the mass spectra generated during the ionization of the single chemical compounds. This process is often done by manual inspection of the raw data and can be time and labor consuming, with the additional concern on the reproducibility of the overall results (Witten and Tibshirani 2013).

**Fig. 1:**
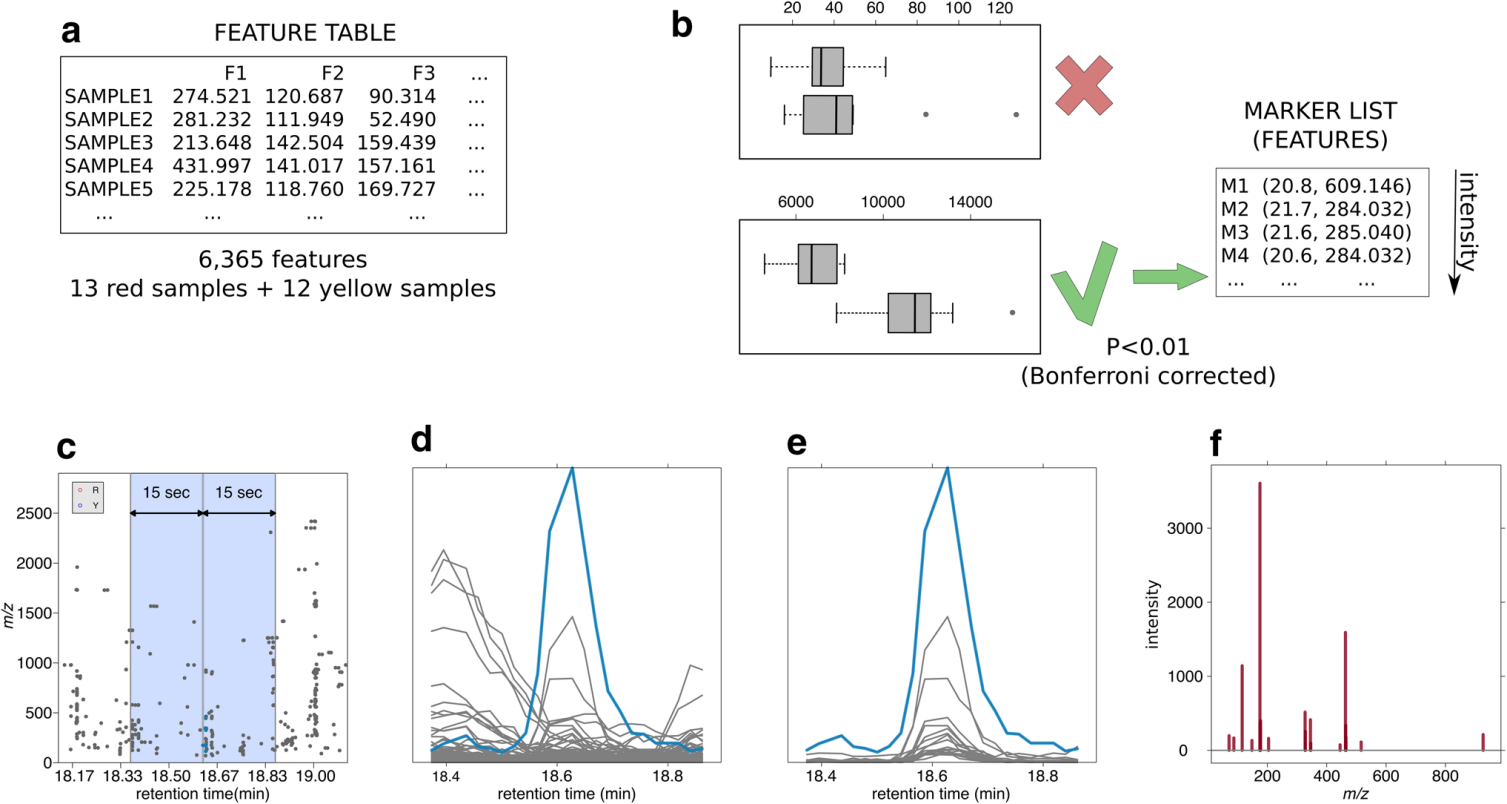
Schema of the main steps of the analysis procedure. **a** Data matrix produced by xcms―CAMERA. **b** Identification of features (within those with intensity greater than 20 cps and *m/z* smaller than 2000) mostly discriminating the two groups, called markers. Threshold on *t* test p-value adjusted with Bonferroni set to 0.01. **c** For each marker, selection of an interesting subset of features lying within a custom time window around the marker retention time. **d** Extraction of the EIC for the subset identified at the previous step. **e** Computation of the correlation between the EICs against the marker EIC and selection of the features having similar chromatographic profile (correlation greater than 0.85). That features will be the candidates (at least 4) for belonging to the same metabolite. **f** Pseudospectrum of putative metabolites

In order to address these issues we developed a new semi-automatic approach, which is outlined in Fig. 1. The aim is to identify the features that are generated in the ionization of each metabolite and to build its pseudospectrum (Fig. 1f). The central idea behind the algorithm is that all the features belonging to the same metabolite have a similar peak shape and elute at the same retention time. As a consequence, their extracted ion traces (EICs) in the surroundings of the chromatographic peak are highly correlated and a filter on the Pearson correlation value can be used to select the features belonging to each pseudospectrum. It is important to point out that this is in practice what the analyst is doing during the manual inspection of the experimental raw data. The proposed approach starts from the list of features, which are significantly different from red and yellow varieties (called markers), and then builds a theoretical mass spectrum by looking for the features which are highly correlated with each marker. The Pearson correlation on the EICs is performed in the sample where each marker is more intense. In this way, we compute the EIC correlation in the sample where the reference marker is better measured and, then, more reliable.

In what follows, we provide a detailed description of the application of the algorithm to the untargeted dataset. To identify the most reliable markers from the list of 6365 features, we imposed an intensity threshold of 20 cps and focused on *m/z* lower than 2000, reducing the feature list to 2572. Within this shortlist, 114 markers were identified by a *t* test corrected for multiplicity (Bonferroni) at the 0.01 confidence level (Fig. 1b). Considering that the proposed approach is based on a correlation analysis among the extracted ion traces, we discarded seven markers^1^ not showing a well-behaved chromatographic peak (see Figure S2). Considering that the width of the chromatographic peak is compound dependent, the marker EICs were visually inspected to identify the optimal retention time window around each chromatographic peak (Fig. 1c), this step was performed to limit the contribution of off-peak signals to the overall Pearson correlation value. Correlation analysis was then performed between the extracted ion trace of each marker and the ones of a subset of the initial 6365 eluting in the vicinity of the reference peak (Fig. 1e). Ions having a correlation higher than 0.85 with each biomarker were used to construct the pseudospectra, we also decided to focus on pseudospectra composed of at least four ions, in order to be able to get some structural insight from the full scan MS data. The described procedure was applied to all the marker ions starting from the most intense one and discarding the markers, which were already attributed to another pseudospectrum. At the end of the analysis, the list of the initial 107 biomarkers resulted in 24 theoretical pseudospectra (for details see Supplementary Material 1). We selected one of the pseudospectra to validate the described approach: it contains one of the most prominent features showing higher concentration in yellow raspberries, detected at *m/z* 934.071 and retention time 20.8 min. The set of *m/z* values 934.071, 933.067, 1870.165, 633.073, 300.999 belonging to the theoretical pseudospectrum were recognized as very characteristic masses of one of the major ellagitannins in raspberry fruit, sanguiin H-6 (PubChem CID: 16130897) (Gasperotti et al. 2010). This annotation was confirmed by the injection of pure standard, which in this method elutes at the same RT, so this was a 1st level of annotation (Sumner et al. 2007). The pseudospectrum masses obtained semi automatically by the pipeline shows a very good match (77 % of the peaks of the pseudospectrum were confirmed with 0.01 tolerance) with the ion masses present in the pure standard of sanguiin H-6 (Fig. S1).

All the analysis were performed using custom R scripts relying on several open source packages: stats (R Core Team 2015); grid (R Core Team 2015), lattice (Sarkar 2008), RColorBrewer, cowplot, ggplot2 (Wickham 2016) for plots; xcms (Smith et al. 2006) and CAMERA (Kuhl et al. 2012) for the processing of raw MS data; WriteXLS for exporting the pseudospectra.

## Results and discussion

### General

**Fig. 2:**
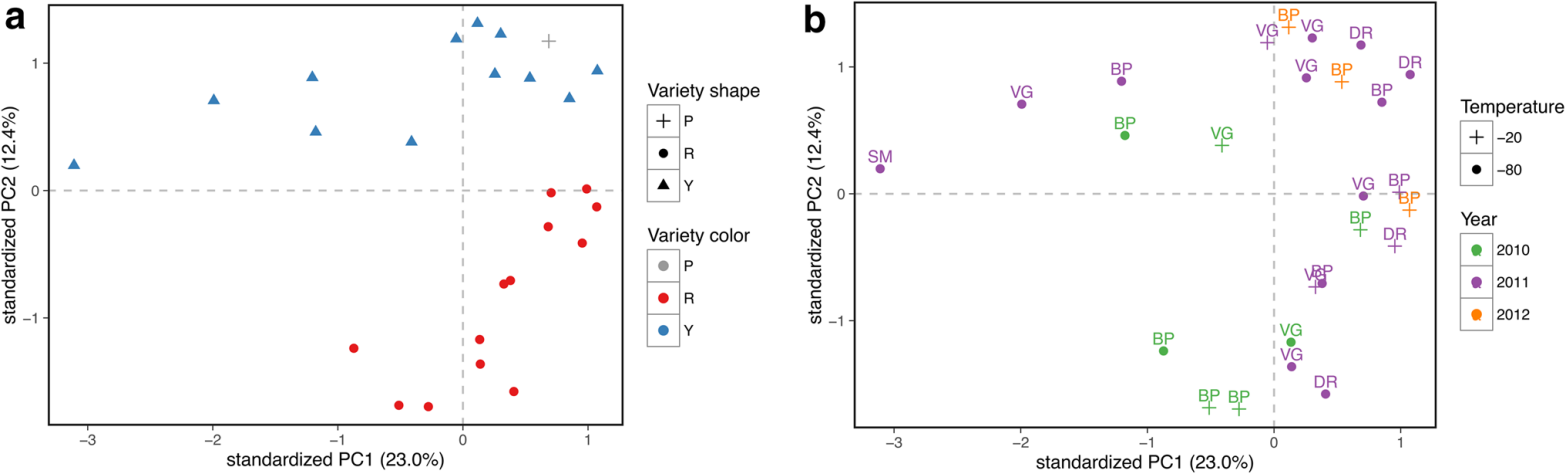
Principal component analysis (*PCA*): representation of the first two principal components of the untargeted dataset (unit variance scaling). **a** The effect of the* berry color* is displayed in the PCA. **b** The effect of the main covariates are shown in the PCA plot (see Table S1): the labels indicate the sampling location (Vigalzano (*VG*), Berry Plant (*BP*), Julius Kühn-Institute (*DR*)), the point style the temperature and the* color* the sampling year

The results of the Principal Components Analysis (PCA) of the untargeted dataset are displayed in Fig. 2. As can be expected for such a complex dataset, the first two principal components are accounting only for the 35 % of the overall variability; however, also in this partial representation of the data space, the separation between yellow and red varieties is clear (Fig. 2a). PCA was also used to check the importance of other characteristics of the samples on the overall variability (Fig. 2b). The figure clearly indicates that neither the origin of the samples, nor the year of collection, nor the storage temperature are separated in the first two principal components and not constitute relevant confounding effects in the yellow/red comparison.

**Fig. 3:**
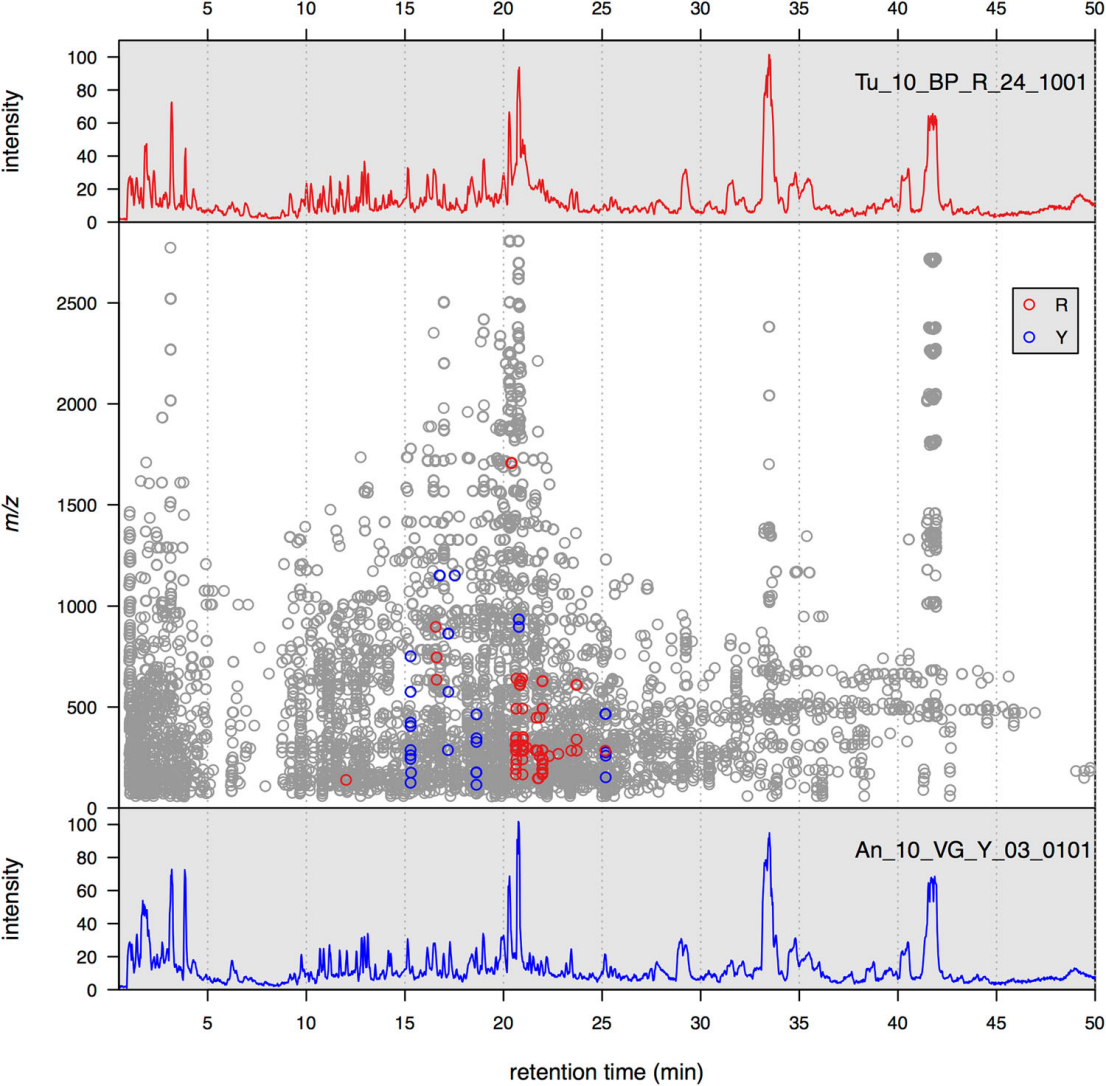
*Middle panel* Features (markers) found to be significantly different between* red* and* yellow* fruits. *Red* features having median intensity higher in* red *raspberries. *Blue* features having median intensity higher in the* yellow* raspberries. *Gray* remaining features. *Top plot* Example of BPI for one red sample (named Tu_10_BP_R_24_1001). *Bottom plot* Example of BPI for one* yellow* sample (named An_10_VG_Y_03_0101)

**Fig. 4:**
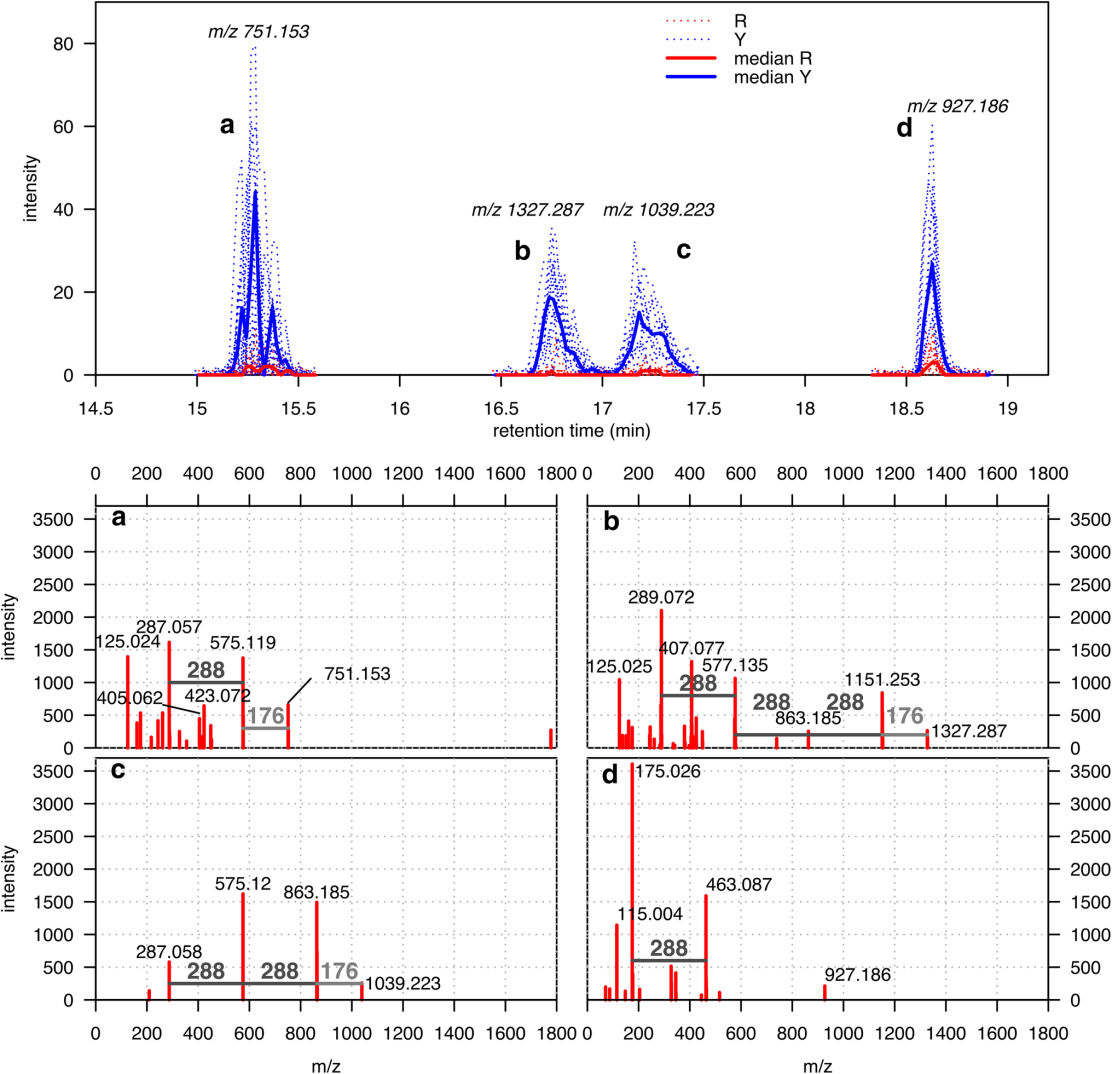
*Top plot* extracted ion traces for putative biomarkers producing pseudospectra (**a–d**). The EICs were extracted in correspondence of the ion which could be associated with the molecular ion, namely 751.153 (**a**), 1327.287 (**b**), 1039.223 (**c**), 927.186 (**d**). *Bottom panel* pseudospectra for the markers **a** 15.3 min, **b** 16.8 min, **c** 17.2 min, **d** 18.6 min. The common losses of 288 and 176 are highlighted in the plots

The typical results of the UPLC–TOF-MS analysis of raspberry extracts are shown in Fig. 3. The base peak chromatograms for a red (top) and yellow (bottom) sample are presented in the uppermost and lowermost panels. The middle panel displays the map of all the 6365 features detected by xcms in the rt-*m/z* plane. With this type of representation, the complexity of the samples is evident, with numerous ions found at different *m/z* values for each rt. The 107 markers, i.e. features found to be significantly different between red and yellow raspberries, elute between 12 and a little over 25 min. A table containing the list of markers can be found in supplementary file markerSumary.xls. In Fig. 3 the features colored in red have median intensity higher in red raspberries. As expected most (83 %) of these features elute between 20 and 22 min, and are characterized by *m/z* values typically observed in the ionization of anthocyanins. Cyanidin 3-*O-*sophoroside (PubChem CID: 44256720) is indeed expected to produce [M − 2H]^−^ and [M − 2H + H_2_O]^−^ ions in negative ion mode (Sun et al. 2012), with *m/z* values of 609.1456 and 627.1561. The theoretical *m/z* of the cyanidin aglycone (A) [A − 2H]^−^ generated by the loss of the sugar moiety is expected at *m/z* 285.0399. Ions with these *m/z* values as well as those of the corresponding isotopes can be seen to be higher in red raspberries (See Supplementary Material 1). It is important to point out that the chromatographic conditions are not optimal for the analysis of anthocyanins, since the low amount of acid in the chromatographic solvents results in very broad anthocyanin peaks, as can be seen in Fig. 4. Considering that the anthocyanin composition of red and yellow raspberries has been widely studied, in the following we will focus on the variables showing higher intensity in yellow berries.

### Compounds higher in yellow raspberries: procyanidin related compounds

Features showing higher intensities in yellow raspberries than in red ones are listed in Supplementary Material 1. We are going to focus on a group of them showing pseudospectra with similar characteristics, which are displayed in Fig. 4 together with their representative extracted ion traces. The top panel of Fig. 4 shows the elution profiles of the *m/z*, which are most likely associated with the intact molecules. For these ionic species, clear chromatographic peaks eluting at 15.3, 16.8, 17.2 and 18.6 min are visible for the yellow raspberries, while they are almost undetectable in all red berries. Considering the characteristics of our sample set, the presence of relatively higher amounts of the associated compounds in yellow berries seems to be common regardless of variety, location or collection year.

The pseudospectra of the first three substances show several common characteristics. The pseudospectrum in Fig. 4a contains ions at *m/z* 575.119 and 751.153, which can be related by a loss of 176.034. In this pseudospectrum one of the most prominent ions is detected at *m/z* 287.057 and can be generated from the 575.119 ion by a loss of 288.062. The second compound (Fig. 4b) shows a similar ionization pattern: the loss of 176.034 now relates *m/z* 1327.287 and 1151.253, while the one of 288.068 relates 1151.253 and 863.185. A similar pattern, with the losses of 288.062 and 176.038, is present also in the third compound (Fig. 4c), which relates respectively the ions at *m/z* 287.058 and 575.120, and those at *m/z* 863.185 and 1039.223.

The pseudospectrum displayed in Fig. 4b shows also two important ions at *m/z* 289.072 (Fig. S3) and 577.135 (Fig. S4), but their intensity turned out to be comparable in red and yellow raspberries. These two ions, then, belong most likely to coeluting compounds. The presence of such “false positives” is a side effect of the use of the correlation among the extracted ion traces to reconstruct the compound pseudospectra. The similarity between the extracted ion traces, indeed, can also group together ions belonging to co-eluting metabolites which are not showing any difference in intensity between red and yellow sample.

The common characteristics of the pseudospectra a, b, and c suggest that their respective compounds might be related: the ions at 287, 575 and 863 are visible at two or more different retention times, and losses of 288 and 176 are common to all three compounds. On the bases of the observed characteristics, we propose that the group of compounds eluting at 15.3, 16.8, 17.2 min could correspond to A-type procyanidins of different polymerization degree which are schematically represented in Fig. 4a–c, respectively. Thus the structural characterization of these metabolites belongs to the 3rd level of annotation (Sumner et al. 2007).

**Fig. 5:**
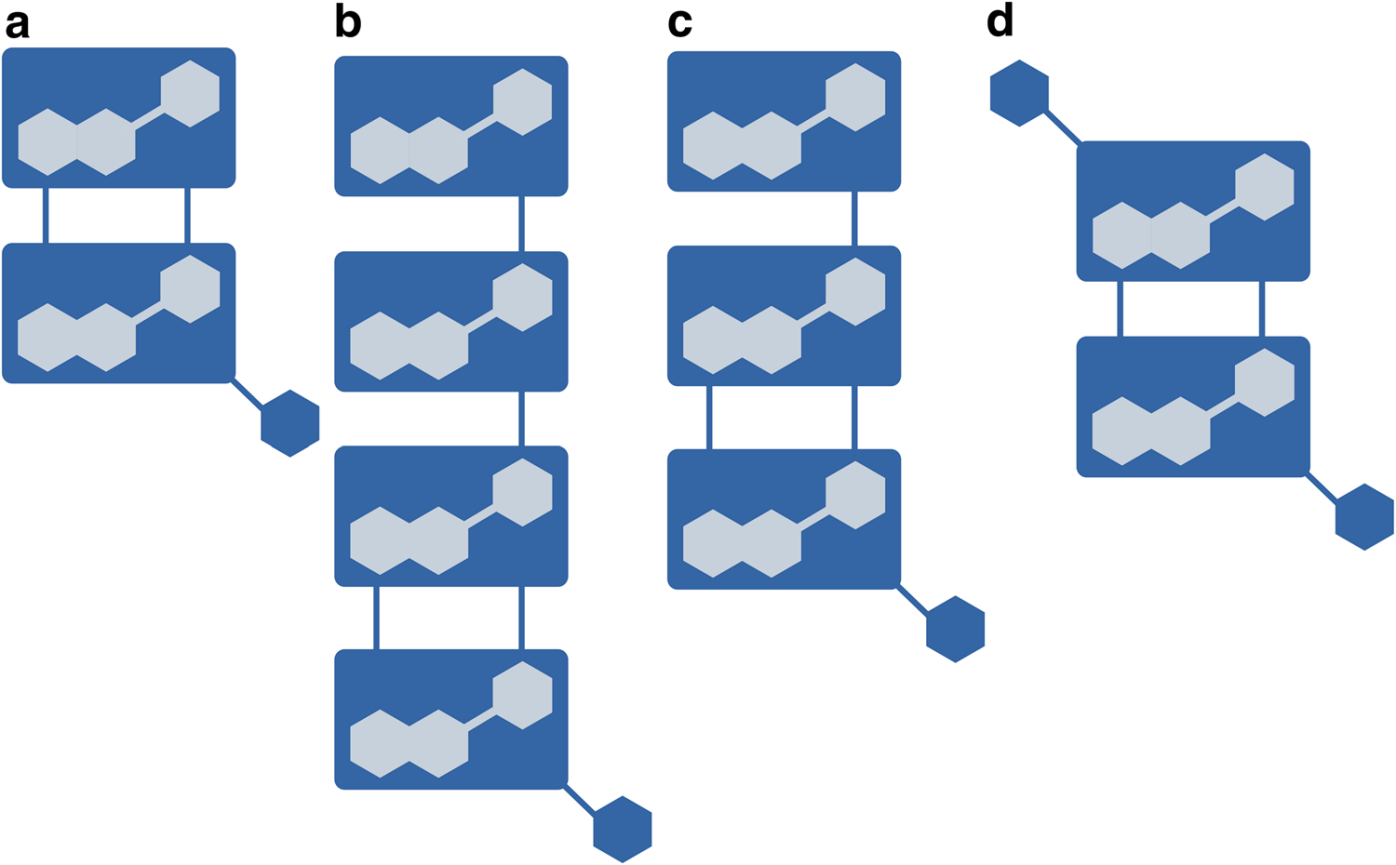
Proposed scheme of the A-type procyanidins corresponding to the pseudospectra in Fig. 4a–d. The *rectangles* represent a catechin or epicatechin unit bound by one or 2 bonds and the *hexagon* the glucuronic acid unit. There is no evidence of the exact location of the glucuronic acid unit and this could be attached to any of the (epi)-catechin units

Raspberries have been described to contain B-type procyanidins (PubChem CID: 21881649), where the monomeric units are linked through C4–C8 or C4–C6 bonds. Procyanidins of type A, which have an extra C2–O–C7 linkage, are less frequent in nature but can be found in peanuts (Tamura et al. 2013), horse-chestnut (Morimoto et al. 1987), cranberries (Toomik et al. 2014), cocoa (Hatano et al. 2002) and plum (Nunes et al. 2008). The presence of the [M – H]^−^ ions in mass spectra of these compounds is common for dimers (theoretical *m/z* 575.1190), trimers (863.1824) and tetramers (1151.2458) (Gu et al. 2003). The fragmentation of an A-type trimer [(epi)Cat-(epi)Cat-A-(epi)Cat)] detected in plums is described in (Gu et al. 2003): such trimer is expected to produce an ion at *m/z* 575.1, since the A-type interflavan linkage of the trimer is located between the middle and base units, and a conjugate ion at *m/z* 287 from the top unit. Conversely, the mass spectrum of the tetramer (epi)Cat-(epi)Cat-(epi)Cat-A-(epi)Cat contain ions at *m/z* 1151, 863 and 575. These fragments can be found in our pseudospectra 4a–c. The recurrent neutral loss of 176 suggests that these compounds could be decorated with a glucuronide molecules as described by Tala et al. 2013 (Gu et al. 2003) and glycosides of A-type procyanidin have been previously found in cacao liquor (Hatano et al. 2002). A scheme of the structures proposed is shown in Fig. 5. Namely, the compound whose pseudospectrum is displayed in Fig. 4a could correspond to an A-type dimer with a glucuronic acid molecule (epi)Cat-A-(epi)Cat-GlcA, the one in Fig. 4b a tetramer (epi)Cat-(epi)Cat-(epi)Cat-A-(epi)Cat-GlcA and the one in Fig. 4c a trimer (epi)Cat-(epi)Cat-A-(epi)Cat-GlcA.

Further indication that this might be the case comes from the presence of the ions at *m/z* 423.072 and 405.062 in the pseudospectra displayed in Fig. 4a: the first ion has been described to occur in A-type dimers, resulting from a retro-Diels–Alder fission of the heterocyclic ring system, and the second corresponds to the sequential water elimination (Karonen et al. 2004; Li et al. 2012).

Lastly, the pseudospectrum displayed in Fig. 4d shows ions at *m/z* 463.087 and 175.026, which can be again linked by a loss of 288.061. At odds with the previous species, a potential loss of 176 could not be seen but the 175.026 fragment is quite clear. It is also visible a *m/z* peak at 927.186. The loss between this and 463.087 is 464.099 *m/z*, indicating that this compound could be a procyanidin A-type dimer with two glucuronic acid moieties, as proposed in Fig. 5d). Also the structural characterization confidence of this metabolite belongs to 3rd level (Sumner et al. 2007).

In order to confirm our hypothesis on the presence of A-type procyanidins, we performed MS/MS experiments on their putative parent ions (*m/z* 751, 1039, 1327 and 927). This assay is also expected to validate the pseudospectra obtained by our semi-automatic analysis pipeline on the full scan dataset. Yellow raspberry extracts were injected on the UPLC-Q-Tof-MS, the parent ions were selected by the quadrupole mass filter at their respective elution times and fragmented in the collision cell. The mass spectra are displayed in Fig. S5. On the overall, 84 % of the peaks assigned by the pipeline to the four metabolites were confirmed experimentally. Looking to the individual species, the confirmed assignments are 100 % for a, 17 % for b, 86 % for c, and 67 % for d. The level of matching is low in the case of b, but also here the most characteristic ions of higher mass show a good match. Remember also that the presence of possible co-eluting compounds in the case of b has been already hypothesized.

**Fig. 6:**
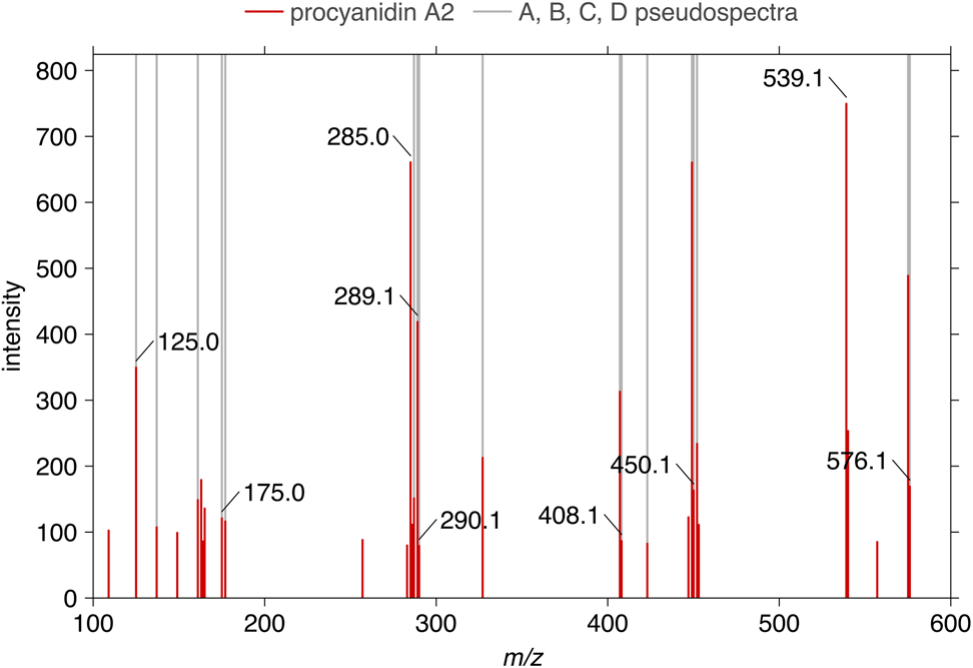
Highlight of the peaks belonging to the pseudospectra* a*,* b*,* c*, and* d* represented in Fig. 4 (*gray lines*) on the standard injection of the procyanidin A2 dimer (*red lines*). Low intensity peaks were filtered (lower than 10 % intensity)

As a further confirmation of our results, the authentic standard of procyanidin A2 has also been injected and its molecular ion was fragmented in the same conditions. The resulting mass spectrum, normalized to the maximum intensity ion, is displayed in Fig. 6. By considering only the most prominent ions (intensity cutoff at 10 %), the spectrum is composed of 31 ions, 17 of them (55 %) are also present in our pseudospectra. Among the matching ions is possible to find those at *m/z* 125.0, 175.0, 287.1, 289.1, 407.1, and of course 575.1. The high number of ions in common between automatically generated pseudospectra and MS/MS experiment confirms the adequacy of our semi-automatic pipeline. It is worth noting that some of the non-matching ions could be originated in the ionization of procyanidins of higher degrees of polymerization and by the presence of glucuronic acid units.

Yellow raspberries differ from their red relatives because of a block in the anthocyanin biosynthesis. The presence of higher amounts of a family of compounds that share characteristics with A-type proanthocyanidins is quite interesting. The building blocks of proanthocyanidins, are flavan-3-ols, which share most of their biosynthetic pathway with anthocyanins. Leucoanthocyanidins can be reduced to *trans*-flavan-3-ols (e.g. (+)-catechin) by the action of leucoanthocyanidin reductase (LAR), or converted into *cis*-flavan-3-ols (e.g. (−)-epicatechin) by the action of anthocyanidin synthase (ANS) and anthocyanidin reductase (ANR): ANS oxidizes leucoanthocyanidins forming anthocyanidins which are then reduced by ANR forming *cis*-flavan-3-ols. The synthesis of these monomers occurs in the cytosol. Epicatechin can be transformed into epicatechin 3′-*O*-glucoside by a glycosyltransferase and thought to be transported by a vacuolar multidrug and toxic compound extrusion (MATE) transporter. The final condensation into oligomers in vivo still remains a mystery (Pang et al. 2013).

Expressing LAR from *Camellia sinensis* in tobacco plants lead to the accumulation of high amounts of precursors (Pang et al. 2013). The authors found not only catechin but also epicatechin and epicatechin glucoside in the plants, suggesting an epimerization activity of this enzyme. This has also been confirmed in another study in which the authors over-expressed LAR from cocoa plant in an *Arabidopsis* mutant containing no ANS (Liu et al. 2013): the authors found increased amounts of catechin but also epicatechin, which in the absence of ANS should not have been there according to the above established pathway. The authors then tested this LAR for dual reductase–epimerase activity in vitro, but only found the reduction product catechin, and no epimerase product epicatechin. Several hypotheses were proposed for the appearance of epicatechin in an ANS mutant, including the presence of an epimerase (other than LAR) in vivo converting catechin to epicatechin, or the racemization of catechin by polymerization to procyanidins and non-stereospecific depolymerization. The latter theory had been previously advanced (Szankowski et al. 2009), after finding that silencing ANS in apple leads to an expected drastic reduction in anthocyanins but also to significant increases in epicatechin and decreases in epicatechin derived polymers. Our findings of an increased family of compounds, likely corresponding to polymeric proanthocyanidins, could indicate an attempt of the plant to increase the synthesis of polymers either as a means of detoxifying excess precursor, or to maintain the levels of epicatechin in the cell: by increasing the polymerization, it could be possible to obtain epicatechin even in the absence of ANS.

In conclusion, it is important to remark that the low intensity of the signals of these procyanidin polymers detected in our experiment does not necessarily mean that they are present in low amounts in the fruit body: procyanidin polymers (also of higher order) can indeed be lost during sample preparation/extraction.

## Concluding remarks

In the present study we have performed untargeted metabolomics analysis, and compared all the metabolic features visible by RP-LC–MS between red and yellow fruits. Apart from the lack of anthocyanidins, which has been well described, we have found several features that were significantly higher in yellow raspberries. To perform our investigation we designed a semi automatic data analysis pipeline able to extract the pseudospectra of the biomarkers directly from the full-scan dataset. Based on the exact mass and pseudospectra data, we made the preliminary proposal that these compounds are different oligomers of A-type procyanidins, modified with a glucuronic acid. This assumption as well as the pseudospectra obtained using the semiautomatic method, was further validated by MS/MS of the heavier ions.

Without the use of untargeted metabolomics and a proper automated data analysis pipeline it would be almost impossible to find these compounds, which are not present in massive amounts among all the other features. The fact that the compounds have a relatively uncommon double linkage and glycosidic modification would make them even more difficult to find, even if one would be looking for procyanidin polymers. In these conditions, the proposed data analysis strategy maximizes the information, which can be extracted from the complex dataset producing evidences that can be used to identify the compounds corresponding to the most relevant features.

Further work would be required to fully characterize the putative procyanidins and confirm their nature. However, it is still interesting that yellow raspberries show a set of structurally related compounds that seem to be absent in red raspberries. This finding opens many doors in proanthocyanidin research and our colleagues working in the field might be interested in keeping an eye open for the presence of such compounds.

## Electronic supplementary material

Below is the link to the electronic supplementary material.

Supplementary material 1 (XLS 18 kb)

Supplementary material 2 (PDF 934 kb)

## Footnotes

1 *We removed the features (20.2, 284.033), (21.9,594.154), (21.9,593.152), (21.4,149.025), (21.6,241.051), (22,199.041), (22.7,299.128).*
