# Supplementary material for: Discovery of A-type procyanidin dimers in yellow raspberries by untargeted metabolomics and correlation based data analysis
Source: Metabolomics. 2016 Aug 8;12(9):144. doi: 10.1007/s11306-016-1090-x (PMC5047924; doi:10.1007/s11306-016-1090-x)
Supplement: Supplementary file 2 — Supplementary material 2 (PDF 934 kb) [file 11306_2016_1090_MOESM2_ESM.pdf]

Discovery of A-type procyanidin dimers in yellow raspberries by untargeted metabolomics and correlation based data analysis

Elisabete Carvalho§, Pietro Franceschi§*, Antje Feller, Lorena Herrera, Luisa Palmieri, Panagiotis Arapitsas, Samantha Riccadonna, Stefan Martens

Supplementary Material

Table S1
Sample list.

Variety	Year	Location	Colour	°C
Orange Marie	2011	DR	Pink	-80
Autumn Bliss	2011	VG	Red	-80
Autumn bliss	2011	DR	Red	-20
BP1	2010	BP	Red	-20
Heritage	2011	VG	Red	-20
Polka	2010	BP	Red	-80
Popiel	2010	VG	Red	-80
Sugana Red	2010	BP	Red	-20
Sugana Red	2011	BP	Red	-20
Sugana Red	2012	BP	Red	-20
Tulameen	2010	BP	Red	-20
Tulameen	2011	VG	Red	-80
Tulameen	2011	BP	Red	-80
Tulameen	2011	DR	Red	-80
Alpen Gold	2011	SM	Yellow	-80
Alpen Gold	2011	BP	Yellow	-80
Alpen Gold	2012	BP	Yellow	-20

§These authors contributed equally
E. Carvalho, P. Franceschi, A. Feller, L. Herrera, L. Palmieri, P. Arapitsas, S. Riccadonna, S. Martens
Research and Innovation Centre
Fondazione Edmund Mach (FEM)
Via E. Mach 1, 38010 San Michele all'Adige, Italy
E-mail: pietro.franceschi@fmach.it

Anne	2011	VG	Yellow	-80
Anne	2010	VG	Yellow	-20
Fall Gold	2011	VG	Yellow	-80
Golden Queen	2011	VG	Yellow	-20
Juan de Metz	2011	VG	Yellow	-80
Lumina	2011	DR	Yellow	-80
Sugana Giallo	2010	BP	Yellow	-80
Sugana Giallo	2012	BP	Yellow	-20
Sugana Giallo	2011	BP	Yellow	-80

Table S2
Values of the xcms parameters used in the analysis.

Parameter	ppm	peakwidth	bw	minsamp	minfrac	mzwid
Value	30	5 − 60	5	2	0	0.05

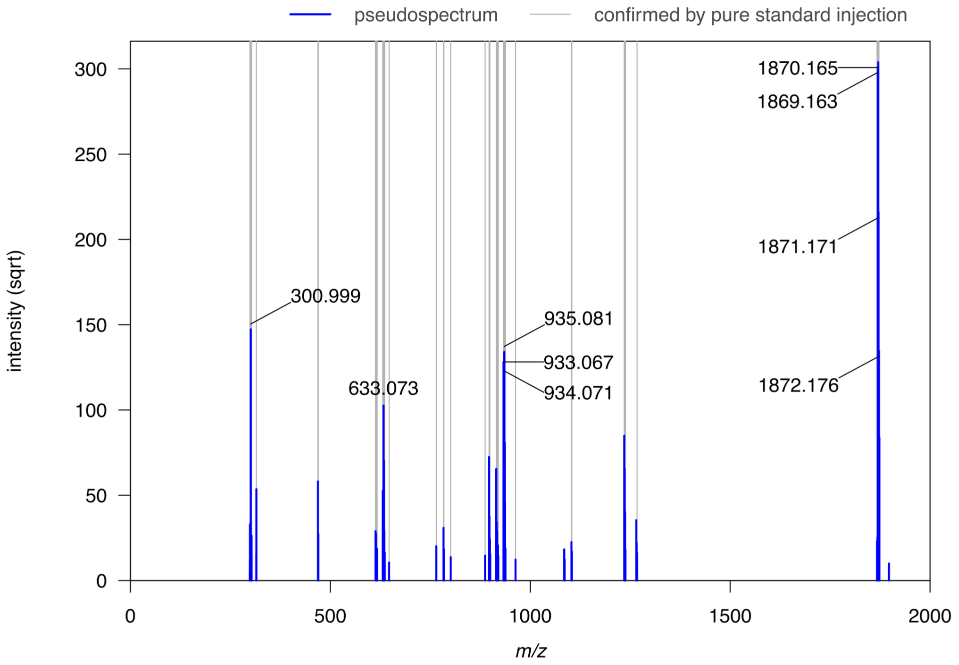

Fig. S1 Data confirmed by MS-MS experiments (77% of the peaks, 0.01 tolerance) for the sanguiin H6 pseudomarker, shown in Fig. 4c.

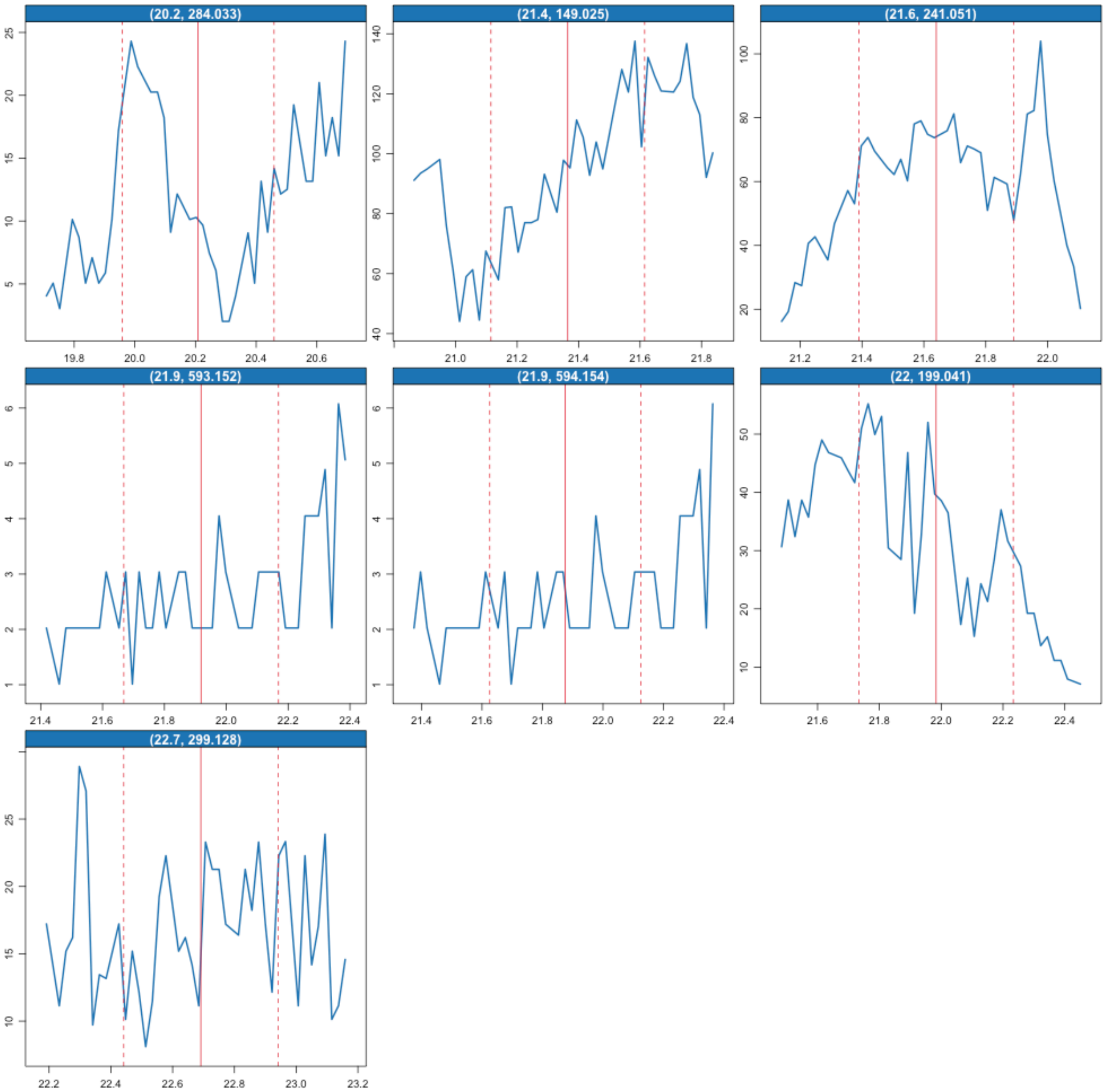

Fig. S2 Markers discarded because they are not showing a well-behaved chromatographic peak. The red solid red line indicates the peak retention time and the red dotted line the 15 secs interval around the peak.

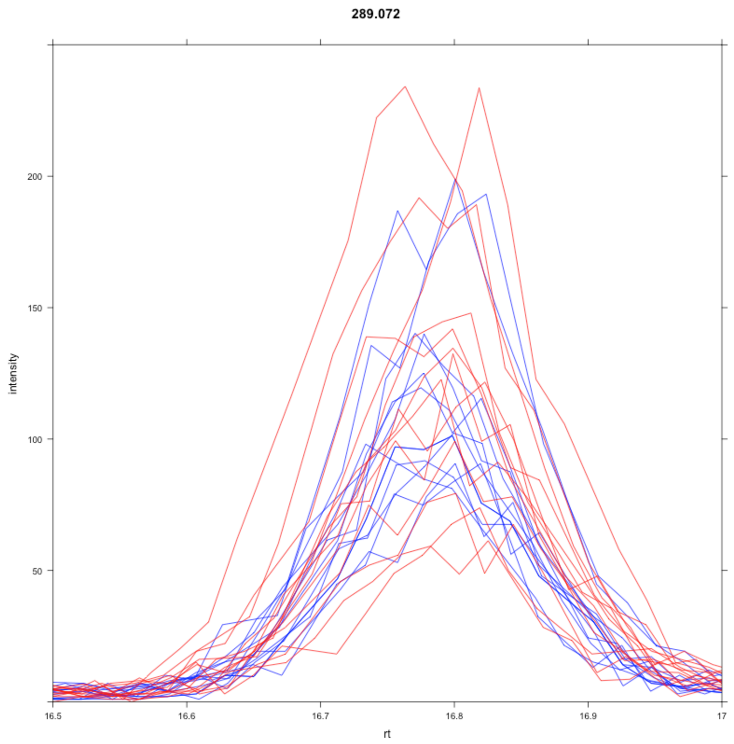

Fig. S3 EICs for the ion 289.072 ± 0.002, which belongs to the pseudospectrum represented in Figure 5b. Blue lines: yellow raspberry samples. Red lines: red raspberry samples.

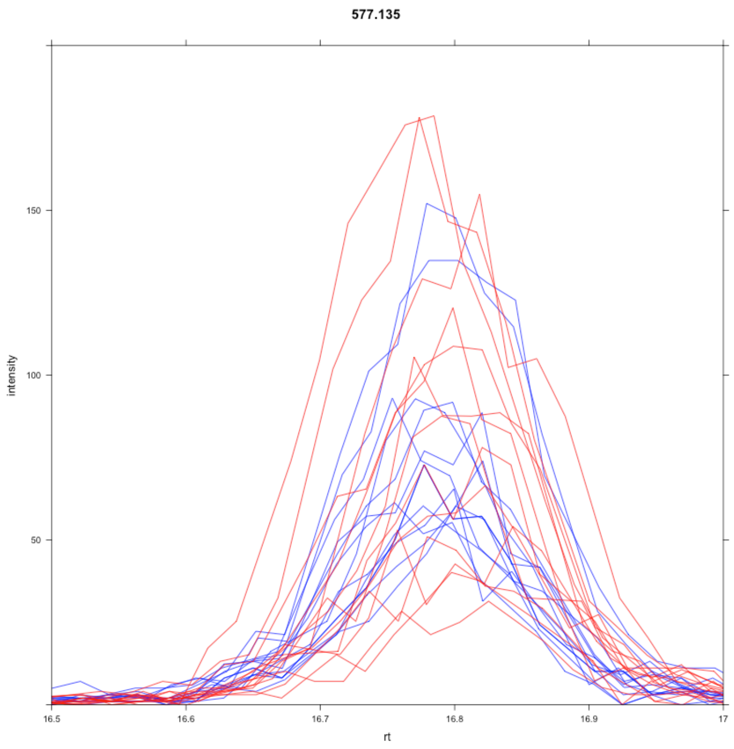

Fig. S4 EICs for the ion 577.135 ± 0.002, which belongs to the pseudospectrum represented in Figure 5b. Blue lines: yellow raspberry samples. Red lines: red raspberry samples.

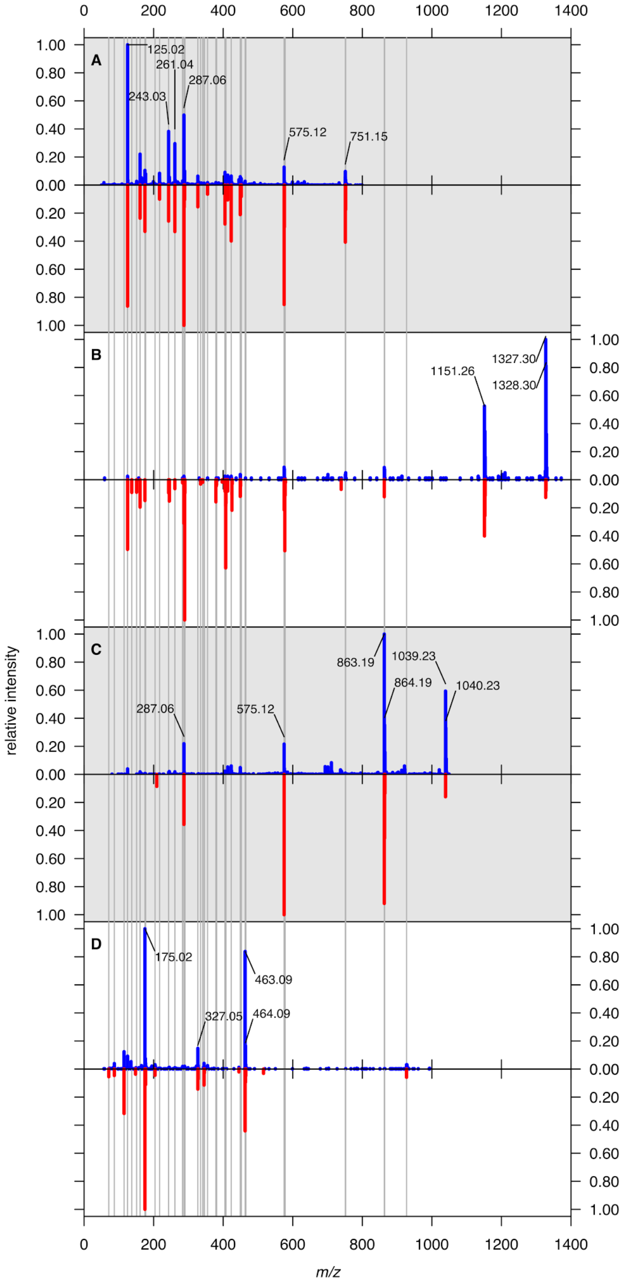

Fig. S6 Top-blue. Spectra from MS/MS experiments obtained by ionization of the following parent ions: (A) m/z 751.2; (B) m/z 1327.3; (C) m/z 1039.2; (D) m/z 927.2. Bottom-red. Pseudospectra obtained by our correlation-based pipeline (as reported in Figure 5 (A)-(D)). Gray lines: peaks of the pseudospectra confirmed by MS/MS experiment.
